# Supplementary material for: Ethylene Inhibits Anthocyanin Biosynthesis by Repressing the R2R3-MYB Regulator SlAN2-like in Tomato
Source: Int J Mol Sci. 2022 Jul 11;23(14):7648. doi: 10.3390/ijms23147648 (PMC9316371; doi:10.3390/ijms23147648)
Supplement: Supplementary file 1 [file ijms-23-07648-s001.zip › Supporting Information.pdf]

Appendix
Supporting Information

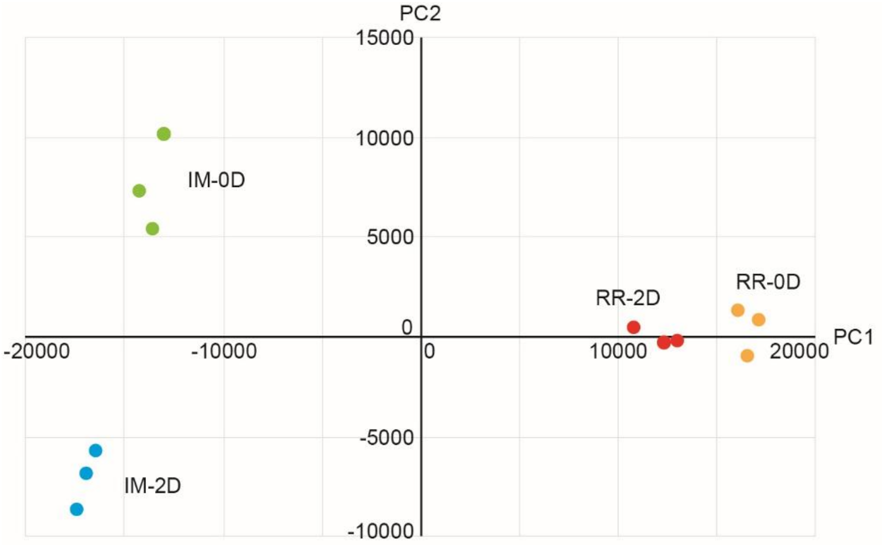

Figure S1 Principle component analysis (PCA) of the RNA-seq datasets

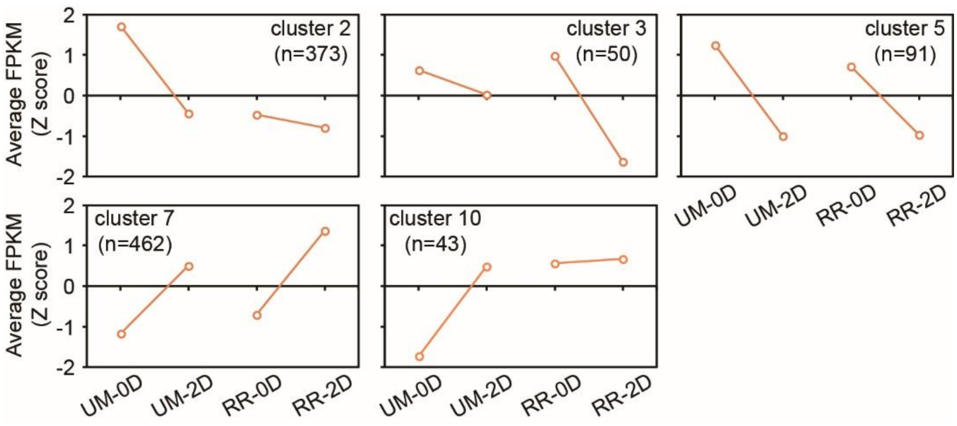

Figure S2 Co-expression patterns of the DEGs identified by pairwise comparison of the four RNA-seq treatments. The y axis indicates the normalized FPKM (Z score).

Table S1. Primer used for qRT-PCR analysis

Gene name	Gene ID	Primer sequence (5'-3')	
		**Forward primer**	**Forward primer**
4CL	*Solyc06g068650*	TTCACCTCAGGGATATCATGCTCATC	CATGTGAACAAGGTGAAGGATTATGC
ANS	*Solyc08g080040*	GAACTAGCACTTGGCGTCGAA	TTGCAAGCCAGGCACCATA
CHI	*Solyc05g010320*	CGGCGCAGGGAATAGAGGTTT	CCGTCAAGGGCAAGATCATAGTCAC
DFR	*Solyc02g085020*	AGTCCAAGGATCCAGAGAACGAAGTA	TGGACATCAAGAGTTCCAGCAGAT
F3H	*Solyc02g083860*	GTGAAAAGTTGATGGATTTGGC	GTAATGGTTCCTGGATCGGTGTGT
F3'5'H	*Solyc11g066580*	GGTACATGTGGGATGGTTGTTGC	ACTTCCAACGTGGTCCATAGGG
SlACTIN	*Solyc03g078400*	GGGATGGAGAAGTTTGGTGGTGG	CTTCGACCAAGGGATGGTGTAGC
SlAN1	*Solyc09g065100*	CTAAGAGTGCCCGCATACAGAC	ATCCGAAGTGGAGTGCTCAGATA
SlAN2-like	*Solyc10g086290*	CAAGCTTCTAGGCAATAGATGGTCACT	TTGGTCGTGGTCGTAGTATAGTGC
SlHY5	*Solyc08g061130*	TCTATTGCCGCTAGTTCACTAC	GGCACTCTTCTGATCTCATCAT

Primer sequences were taken from our previous study (Cao *et al.*, 2017).

Table S2. Summary of the transcriptome data.

Sample	Raw reads	Unique mapped reads
IM-2D-1	26,687,545	22,359,437
IM-2D-2	23,249,026	19,525,114
IM-2D-3	22,911,485	18,981,050
RR-2D-1	23,699,982	20,168,114
RR-2D-2	24,725,132	20,955,826
RR-2D-3	23,641,723	20,076,151
IM-0D-1	25,668,053	21,570,979
IM-0D-2	25,685,396	21,360,007
IM-0D-3	25,613,901	21,393,245
RR-0D-1	22,959,477	19,475,948
RR-0D-2	21,924,746	18,555,827
RR-0D-3	22,655,264	19,221,291
